# Influence of HIV infection on the clinical presentation and outcome of adults with acute community-acquired pneumonia in Yaounde, Cameroon: a retrospective hospital-based study

**DOI:** 10.1186/1471-2466-12-46

**Published:** 2012-08-30

**Authors:** Eric Walter Pefura Yone, Adamou Dodo Balkissou, André Pascal Kengne, Christopher Kuaban

**Affiliations:** 1 Department of Internal Medicine and Subspecialties, Faculty of Medicine and Biomedical Sciences, University of Yaounde 1, Yaounde, Cameroon; 2 Service of Pneumology, Yaounde Jamot Hospital, BP: 4021, Yaoundé, Cameroun; 3 South African Medical research Council & University of Cape Town, Cape Town, South Africa

**Keywords:** Community-acquired pneumonia, HIV infection, Hospitalisation, Outcomes, Cameroon

## Abstract

**Background:**

The impact of HIV infection on the evolution of acute community-acquired pneumonia (CAP) is still controversial. The aim of this study was to investigate possible differences in the clinical presentation and in-hospital outcomes of patients with CAP with and without HIV infection in a specialised service in Yaounde.

**Methods:**

Medical files of 106 patients (51 men) aged 15 years and above, admitted to the Pneumology service of the Yaounde Jamot Hospital between January 2008 and May 2012, were retrospectively studied.

**Results:**

Sixty-two (58.5%) patients were HIV infected. The median age of all patients was 40 years (interquartile range: 31.75-53) and there was no difference in the clinical and radiological profile of patients with and without HIV infection. The median leukocyte count (interquartile range) was 14,600/mm^3^ (10,900-20,600) and 10,450/mm^3^ (6,400-16,850) respectively in HIV negative and HIV positive patients (p = 0.002). Median haemoglobin level (interquartile range) was 10.8 g/dl (8.9-12) in HIV negative and 9.7 g/dl (8–11.6) in HIV positive patients (p = 0.025). In-hospital treatment failure on third day (39.5% vs. 25.5.1%, p = 0.137) and mortality rates (9% vs. 14.5%, p = 0.401) were similar between HIV negative and HIV positive patients.

**Conclusion:**

Clinical and radiological features as well as response to treatment and in hospital fatal outcomes are similar in adult patients hospitalised with acute community-acquired pneumonia in Yaounde. In contrast, HIV infected patients tend to be more anaemic and have lower white cell counts than HIV negative patients. Larger prospective studies are needed to consolidate these findings.

## Background

Acute community-acquired pneumonia (CAP) is a common cause of morbidity and mortality in sub-Saharan Africa (SSA)[1-3], where it is highly frequent in people with HIV infection [4]. The possible effects of HIV infection on the evolution of CAP are still controversial. For instance, whether HIV infection negatively impacts on fatal outcome in patients with CAP is still uncertain [1,3,5-7], justifying at times the exclusion of HIV patients from trials on CAP [6]. In spite of the proven efficacy of empirical antibiotic treatment on CAP in people with HIV [8-10], the management of the condition in SSA is still compromised by the lack of bacteriological investigations. Moreover, the fear of pejorative evolution often leads to frequent hospitalisation of CAP patients with HIV, even in the absence of classical clinical signs of severity of the disease [6].

In Cameroon, a study investigating the effects of HIV infection of CAP 15 years ago, reported frequent bacteraemia in patients with HIV [1]. In the context of the growing population of individuals with HIV infection in SSA in general, the current report aims to update the knowledge on the potential effects of HIV infection on CAP by investigating differences if any in the clinical presentation and in-hospital outcomes of CAP patients with and without HIV infection in urban setting in Cameroon.

### Materials and methods

#### Study setting and participants

The study was undertaken in the pneumology service of the Yaounde Jamot Hospital (YJH), which has been described in details elsewhere [11,12]. In brief, YJH serves a referral centre for chest disease for the capital city of Cameroon (Yaounde) and surrounding areas.

CAP in this hospital is diagnosed based on 1) lower respiratory tract infection signs, 2) new alveolar opacity on chest X-ray and 3) the absence of other possible causes of the alveolar opacity [13]. All chest X-rays are read by radiologist and reviewed by treating chest physician. Patients with radiological signs suggestive of other possible causes, or in whom radiological signs do no regress on treatment are prescribed a bronchial fibroscopy to rule out tuberculosis and others opportunistic infections. Upon admission to this hospital, routine evaluation of patients suspected of having CAP received include a clinical examination, chest X-ray (frontal and lateral views), sputum examination for Acid Fast Bacillus (AFB) in patients who can produce a sputum (three series at the beginning of the treatment and three series at the completion of the treatment) and biological workup comprising a full blood count, fasting glycaemia, erythrocyte sedimentation rate and HIV test after informed consent. HIV testing includes detection of anti-HIV 1 and anti-HIV 2 antibodies in the serum with the use of two rapid tests: Determine® HIV ½(Abbot laboratories, Tokyo, Japan) and Immunocomb® II HIV 1 and 2 Bispot (Organics, Courbevoie, France). A patient is classified as HIV positive when the two tests are positive. For discordant tests, a confirmatory western blot test (New Lav Blot, Sanofi diagnostics-Pasteur) is conducted. All HIV-positive patients are started on prophylaxis with cotrimoxazole and those with CD4 lymphocyte counts < 200/mm^3^ or with recurrent pneumonia (at least 2 episodes within a 12 month window) are started on triple antiretroviral therapy free of charge.

On admission to the service, patients are treated empirically with a betalactam antibiotic and treatment is adjusted according to patients’ clinical response. Bacteriological investigations to isolate the microbial aetiology are hardly ever done because of limited resources and lack of diagnostic facilities. Monitoring is based on daily assessment of the clinical profile including body temperature, and chest X-ray on the tenth day from treatment inception in those with favourable treatment course. Favourable response to treatment on the third day is defined by the regression of lower respiratory tract infection signs and particularly thermic defervescence or body temperature normalisation [13]. Treatment failure at this same time point is based on the non-abatement of the fever and worsening of chest signs. Patients with treatment failure are re-assessed for differential diagnoses, loco-regional complications (lung abscess, purulent or parapneumonic pleural effusion), extra-pulmonary infection and bacterial resistance to the initial empirical treatment.

All the patients admitted in the service are indexed by the chief nurse of each hospitalization unit as their arrival in hospitalization registers envisaged for this purpose. For each in-patient, the following elements are consigned in the registers: age, sex, admission and discharge date, results of HIV serology, admission and discharge diagnosis.

## Methods

Recruitment for this study was restricted to patients aged 15 years and above, followed for CAP at any time point during January 2008 through May 2012 (total duration 53 months). Patients’ hospitalisation registers were reviewed to identify those who were hospitalised for CAP and their medical files were then retrieved. For all eligible patients, data were collected on the demographic profile including sex and age, past medical history and comorbidities, and disease course prior to hospital admission. Clinical signs and symptoms recorded included the body temperature (with fever defined as a temperature ≥38°C), cough, chest pain, dyspnoea, haemoptysis, respiratory and heart rates, and condensation syndrome. The CRB-65 score [14] presented by each patient at the admission was noted. Standard chest X-ray data upon admission and subsequently were analysed and the following registered: unilateral or bilateral lung parenchyma lesions and number of lobes involved, presence or absence of pleural effusion. Biological data included the full blood count, HIV test results, and CD4 count for HIV positive patients. Evolution data included those relating to treatment failure at day three following inception, local and regional complications, treatment success and all-cause mortality. Patients with missing data for HIV status and those with missing medical file were excluded. The study protocol was approved by the institutional board of the YJH.

### Statistical methods

Data analysis used SPSS® 17.0.1 (SPSS Inc., Chicago, USA) for Windows. Groups comparison used Pearson *χ*^2^ or Fisher exact test for qualitative variables, and Student *t*-test or non prametric equivalents for quantitative variables as appropriate. A p-value <0.05 was used to characterise statistically significant results.

## Results

### Data available

During the study period, a total of 6098 patients were admitted to the service, among whom 118 (1.9%) patients aged ≥ 15 years had CAP. Medical files were available for all of them except six who were excluded. For those with medical file available, six were excluded for missing data on HIV status. Therefore 106 patients (51 being men) with a median age of 40 years (interquartile range: 31.75-53) were included in the final analysis. To validate the selection process, medical files were also retrieved for a random sample of 100 patients who did not have CAP based on registers. None of them also had CAP based on the diagnosis in the medical file. For those patients included data were missing for few participants on some variables and are reported where relevant.

### Prevalence of HIV infection and treatment

Of the 106 patients included 62 (58.5%) were HIV positive, all being infected with HIV-1 serotype. The median CD4 lymphocyte count in those with HIV infection was 211/mm^3^ (interquartile range: 97.5-331). Twenty-one (33.9%) HIV-infected patients had CD4 count lower than 200/mm^3^. HIV infection had been diagnosed prior hospital admission in 28 (45.2%) of them, and 18 (29%) were on antiretroviral therapy.

### Profile of patients by HIV status upon admission

Clinical, radiological and biological characteristics of HIV positive and HIV negative participants are described in Table 1. The two groups were broadly similar with regard to demographic, past medical history, clinical and radiological profiles. Differences were however apparent in the biological profile where patients with HIV infection had lower haemoglobin levels (9.7 vs. 10.8 g/dl, p = 0.025) and lower total leucocyte count (10,450 vs. 14,600, p = 0.003) than HIV negative patients (Table 1).

**Table 1:** Clinical, radiological and hematologic characteristics of patients with acute community-acquired pneumonia, possibly of bacterial origin in Yaoun de

**Characteristics**	**HIV-negative n=44**	**HIV-positive n=62**	**p-value**
**Sex (Women/Men)**	22/22	33/29	0.743
**Age, years**			
≤ 40, n (%)	21 (47.7)	33 (53.2)	0.577
> 40, n (%)	23 (52.3)	29 (46.8)	
**Chest symptoms**			
Cough, n(%)	43/43(100)	60/61(98.4)	0.999
Expectoration, n (%)	34/42 (81)	54/61 (88.5)	0.284
Chest pain, n (%)	42 (95.5)	57 (91.9)	0.697
Dyspnoea, n (%)	26 (59.1)	30 (48.4)	0.277
Duration of symptoms , median(IQR)	10 (7-17)	11 (5.25-14)	0.948
**CRB65 Score**			
< 2, n (%)	33/40(82.5)	50/59 (84.7)	0.319
≥ 2, n (%)	7/40 (17.5)	9 (15.3)	
**Past medical History**			
Smoking, n (%)	10 (22.7)	13 (21.0)	0.829
Excessive alcohol consumption, n (%)	10/42 (23.8)	14/60 (23.3)	0.956
Other comorbidities, n (%)	7 (15.9)	12(19.4)	0.649
**Radiographic features**			
Single lobe involvement, n (%)	17 (35.5)	30(43.6)	0.319
Multiple lobes involvement, n (%)	27 (64.5)	32(56.4)	0.319
Pleural effusion, n (%)	10 (22.7)	11(17.7)	0.526
**Hematologic features**			
Median leucocytes count, mm^3^ (IQR)	14,600 (10,900-20,600)	10,450 (6,400-16,850)	0.003
Median haemoglobin, g/dl (IQR)	10.8 (8.9-12)	9.7 (8-11.6)	0.025
Mean platelet count, mm^3^ (SD)	302,880 (129,588)	259,550 (140,148)	0.116

IQR, interquartile range; SD, standard deviation.

### Outcomes of patients with and without HIV infection

Non-significantly high rate of treatment failure was recorded in HIV negative, as compared to HIV positive patients (35.5% vs. 25.5%, p = 0.137). Loco-regional complications were also more likely to occur in HIV negative patients (40.1%) than among the HIV positive ones (17.7%) (p = 0.008, Table 2). In both groups, the most frequent complication was purulent pleural effusion, which occurred in 7 (15.9%) HIV negative patients and 5 (8.1%) HIV positive patients. The median duration of hospitalisation (interquartile range) was 13 (10–15) days in HIV negative patients and 11.5 (8–16) in HIV positive patients (p = 0.554, Table 2). Death rate during this period and median time to death were similar between the two groups (both p ≥ 0.268).

**Table 2:** Evolution of patients with acute community-acquired pneumonia, possibly of bacterial origin in Yaounde

**Evolution**	**HIV negative n=44**	**HIV positive n=62**	**p-value**
**Treatment failure on 3**^**rd**^day*, n (%)	17/43 (33.3)	14/55 (17.1)	0.137
**Loco-regional complications**			
None, n (%)	26 (59.1)	51 (82.3)	0.008
Parapneumonic pleural effusion, n (%)	5 (11.4)	3 (4.8)	0.271
Purulent pleural effusion, n (%)	7 (15.9)	5 (8.1)	0.229
Lung abscess, n (%)	6 (13.6)	3 (4.8)	0.158
**Median duration of hospitalisation, days (IQR)**	13 (10-15)	11.5 (8-16)	0.554
**Death, n (%)**	4 (9.0)	9 (14.5)	0.401
**Median time to death, days (IQR)**	0.54 (0.15-6.45)	2 (1-10)	0.268

* One HIV negative and seven HIV positive patients had deceased before 3^rd^ day; IQR, interquartile range.

## Discussion and conclusions

In this study conducted in a major referral hospital for chest diseases in Cameroon, we found a high prevalence of HIV-1 infection among patients admitted for acute community-acquired pneumonia. Half of the time, HIV infection was undiagnosed at the time of clinical presentation with CAP. Among those already diagnosed with HIV infection, about half were on antiretroviral therapy. With three exceptions, the clinical, radiological, biological profiles of patients and outcomes of care for CAP were similar between patients with HIV infection and those without. These exceptions relate to the low haemoglobin level and low total leucocytes counts in HIV positive patients and a high complication rates among HIV negative patients.

The prevalence of HIV infection in our study is about the double of the prevalence rate reported in Cameroon 15 years ago [1] based on 110 patients recruited from three different hospitals, probably mirroring the changing prevalence of HIV in the country between the two study periods. Indeed, available data suggest that HIV prevalence was about 3% at the general population in Cameroon in 1994 [15], and increased to about 5% in 2007 [16]. Otherwise, HIV prevalence in our study was closer to the 58% rate reported by [Horo et al.3] in Ivory Coast .

While the well-known young age of patients with HIV may explain part of the findings, our study suggests that hospitalised patients with CAP in this setting were predominantly young individuals. This may reflect the overall young age of the general population. The equal distribution of women among HIV negative and HIV positive patients suggests that high rates of HIV infections among women in Africa [16] may not necessarily translated into more women with HIV developing CAP. Such a claim however, based on a hospital cohort remains very weak in the context of many uncontrolled sources of biases. The broadly comparable clinical and radiological profiles of patients with and without HIV infection found in our study is in agreement with existing reports[1,3]. Of note, the disease severity as assessed by the CRB-65 score [14] was unaffected by HIV status. Differences found in the haematological profile were largely expected. For instance, it is well known that anaemia is of multifactorial causes in the context of HIV infection, and is not necessarily correlated with immune-depression and presence of opportunistic infections [17,18].

The comparable evolution and outcomes of patients regardless of their status for HIV in our sample is largely in agreement with earlier report from the same setting [1]. The high prevalence of loco-regional complications among HIV negative participants in this study is possibly related to a high prevalence of infections due to necrotizing micro-organisms in this subgroup. Indeed, in a recent study on purulent pleural effusions in the same setting, we found that *Staphylococcus aureus* micro-organism was more frequently isolated in HIV negative than in HIV positive patients (23.8% vs. 12.5%), although the difference was not statistically significant, due to the small sample [19]. However, death rate among our much large sample of patients with HIV infection was likely higher than the 7% reported previously based on half the size of our cohort with HIV [1]. Discrepancies are likely due to difference in precisions on the diagnosis of CAP. The former study [1] used microbiological investigations for diagnosis confirmation and accordingly adapting treatments, what our study did not afford. In over half of patients with fatal outcomes in our sample, death occurred with the first 72 h of observation needed to assess the response to treatment. Some would have been the result of inappropriate initial diagnosis or inefficacy of the empirical treatment. In general mortality rate among HIV patients with CAP across published data varies substantially [5,7,8,20-22]. However, available studies on the effect of HIV on fatal outcome in patients with CAP are conflicting with some suggesting a less favourable outcome in patients with severe immune depression [5,21,23], and others suggesting no effect of immune depression on mortality [1,6,7,23]. In a study by [Feldman et al. 24] in HIV positive patients with bateriemic pneumococcal pneumonia, fatality rate was high in patients with severe immune-depression. We instead found no significant difference in death rates among patients CAP patients with HIV according to the severity of the disease (data not shown).

Our study has some limitations including the retrospective nature and as a result data were missing for some participants on some variables including key inclusion variables such as HIV status, HIV viral load and other variables like nutitional and socioeconomic status. In the absence of microbiological investigations, we were unable to have a definitive confirmation of diagnosis. In this context, some patients would have probably been misclassified as having CAP or not, which may have the undesirable effect of biasing the results if occurring in a differential ways. The previous study in this setting suggests using microbiological diagnosis as a standard, physicians diagnosis of pneumonia is accurate in about 50% of cases, and similarly among patients with HIV and those without [1]. The strengths of our study include the relatively larger number of people with HIV and accordingly more power to investigate the effects of prior knowledge of HIV status on clinical presentation and outcome of care for CAP.

In conclusion, HIV infection is highly frequent among patients with CAP in this setting. In major ways however, it seems to be unrelated with key aspects of the disease including clinical presentation, radiological findings, response to empirical antibiotic treatment and fatal outcomes. Therefore similar strategies should be applied to CAP patients regardless of the status for HIV. However, CAP presentation should be used as an opportunity to screen individuals for undiagnosed HIV infection. Larger prospective studies and needed to consolidate these findings.

## Abbreviations

CAP, Acute community-acquired pneumonia; HIV, Human Immunodeficiency Virus; HJY, Yaounde Jamot Hospital; SSA, Sub-Saharan Africa.

## Competing interests

The authors declare that they have no competing interests.

## Authors’ contributions

EWPY conceived and designed the study, performed analysis, interpretated of data and drafted the manuscript, ADB and APK assisted with the design, interpretation of data and the critical review of the manuscript, CK performed critical review of the manuscript. All authors approved the final manuscript.

## Pre-publication history

The pre-publication history for this paper can be accessed here:

http://www.biomedcentral.com/1471-2466/12/46/prepub
